# Interventions to Increase HIV Testing Uptake in Global Settings

**DOI:** 10.1007/s11904-022-00602-4

**Published:** 2022-04-20

**Authors:** Radhika Sundararajan, Matthew Ponticiello, Denis Nansera, Kidola Jeremiah, Winnie Muyindike

**Affiliations:** 1 Department of Emergency Medicine, Weill Cornell Medicine, 525 East 68th Street, M-130, New York, NY 10065 USA; 2 Weill Cornell Center for Global Health, New York, NY USA; 3 Mbarara Regional Referral Hospital, Mbarara, Uganda; 4 Mbarara University of Science and Technology, Mbarara, Uganda; 5 National Institute for Medical Research, Mwanza, Tanzania

**Keywords:** HIV testing, Interventions, Global, Facility-based, Community-based

## Abstract

**Purpose of Review:**

HIV testing is the critical first step to direct people living with HIV (PLWH) to treatment. However, progress is still being made towards the UNAIDS benchmark of 95% of PLWH knowing their status by 2030. Here, we discuss recent interventions to improve HIV testing uptake in global settings.

**Recent Findings:**

Successful facility-based HIV testing interventions involve couples and index testing, partner notification, and offering of incentives. Community-based interventions such as home-based self-testing, mobile outreach, and hybrid approaches have improved HIV testing in low-resource settings and among priority populations. Partnerships with trusted community leaders have also increased testing among populations disproportionally impacted by HIV.

**Summary:**

Recent HIV testing interventions span a breadth of facility- and community-based approaches. Continued research is needed to engage men in sub-Saharan Africa, people who inject drugs, and people who avoid biomedical care. Interventions should consider supporting linkage to care for newly diagnosed PLWH.

## Introduction

HIV testing is the crucial first step of the HIV cascade of care [1], and the first of the three goals set by UNAIDS to achieve epidemic control by 2030, where 95% of PLWH will be aware of their status [2]. Accurate and accessible HIV testing is necessary to direct PLWH to treatment and is essential to preventing ongoing transmissions [3]. In addition, being aware of one’s HIV status has been associated with reductions in behaviors with high transmission risk [4], including syringe sharing [5] and inconsistent condom use [6]. Knowing one’s status is also necessary for use of HIV prevention strategies such as pre-exposure (PrEP) [7] and post-exposure prophylaxis (PEP) [8].

In 2021, only 84% of all PLWH were aware of their status [9]. This number is even lower in sub-Saharan Africa, particularly among men and young people [10], as well as globally among certain populations, such as sex workers, men who have sex with men (MSM), people who inject drugs (PWID), and transgender people [11]. Although there are various modalities of HIV testing implemented throughout the world, all have been shown to be cost-effective [12, 13••], even in low-prevalence settings [14]. In this review, we examine recent advancements in HIV testing interventions to improve uptake of HIV testing across global settings.

## Facility-Based Strategies

Facility-based interventions are those that offer HIV testing at formal healthcare facilities such as clinics or hospitals. At healthcare facilities, individuals can receive an HIV test via voluntary counseling and testing or through provider-initiated HIV testing, in which healthcare providers recommend HIV testing as part of routine medical care. In sub-Saharan Africa, provider-initiated testing has been the primary means of identifying new HIV infections [15]. Provider-initiated HIV testing often falls into two categories: opt-out or opt-in. Opt-out HIV testing refers to when HIV testing is already a component of receiving healthcare and requires individuals to request *not* to receive an HIV, whereas opt-in HIV testing refers to the process where patients are offered an HIV test in addition to whatever services they were seeking. Opt-out HIV testing has been implemented in various outpatient and inpatient clinical settings around the world, resulting in increased HIV testing among individuals receiving medical care at clinical facilities [16, 17]. Interestingly, a meta-analysis of studies from high-income countries found no difference in the detection of new HIV infections between the opt-in and opt-out approaches [18]. Despite increasing uptake of HIV testing, these strategies have had suboptimal reach, particularly among men and young people who tend to avoid medical care until they develop advanced symptoms [18–22]. In an analysis of 2019 PEPFAR data from sub-Saharan Africa, men were still only half as likely to receive HIV testing compared with women [23]. As such, additional initiatives have been implemented for patients who present to medical facilities.

### Provider Initiated Counseling and HIV Self Testing

Provider-initiated counseling and HIV self-testing provide those who present to medical facilities the opportunity to test via supervised HIV self-testing. In Malawi, offering oral swab Oraquick HIV self-tests while at medical facilities quadrupled HIV testing uptake compared to provider-initiated testing: 51% of participants completed self-testing compared to 13% for provider-initiated testing [24•]. In this study, self-testing uptake was particularly high among men and adolescents. Another study in Kenya demonstrated that over half of pregnant and post-partum women elected to receive an HIV self-test offered at public health facilities as opposed to traditional HIV blood testing [25]. HIV self-testing may be a feasible strategy to expand HIV testing among those who avoid HIV testing due to fear of needles and phlebotomy.

### Index Testing

Index testing/partner-assisted notification are facility-based strategies endorsed by the World Health Organization (WHO). Index testing involves offering HIV testing to sexual partners, drug use partners, and biological children of PLWH. Using this approach, family testing for biological children of PLWH has been shown to facilitate rapid identification of children with perinatal HIV across sub-Saharan Africa, including in Zimbabwe where 60% of index-linked children were HIV tested [26]. Unfortunately, these procedures have yet to be routinely integrated into health systems [27]. Partner-assisted notification informs individuals that they may be at risk of HIV acquisition following sexual contact or drug use with a person newly diagnosed with HIV [28]. A study in Kenya found that immediate partner notification was more effective than delayed partner notification, where partners were notified after 6 weeks: 67% of partners who were immediately notified underwent HIV testing compared to 13% in the delayed notification group [29]. Partner-assisted notification has been effective in a variety of contexts, including among Indonesian prison populations and their sexual and drug-use partners [30], as well as among PWID in Kazakhstan, Tajikistan, and the Kyrgyz Republic [31]. One modeling study in Kenya demonstrated that partner-assisted notification is both cost-effective and effective in reducing HIV-related mortality [32]. Results from multiple countries in sub-Saharan Africa revealed that index testing and partner-assisted notification were efficient strategies with high case finding potential [33••], particularly in identifying men living with HIV [23].

### Couples Testing

HIV testing among young women has been high in some countries, like Uganda, for example [34], because it has been well-integrated into routine antenatal care. In an effort to improve testing uptake among men and encourage serostatus disclosure between couples, the WHO has recommended couples-based testing, where pregnant women and their partners test for HIV together at prenatal care visits [35]. A study in South Africa found that adding couples-based counseling sessions to couples testing programs improved uptake of HIV testing by 30%, with 42% receiving HIV testing compared to 12% in the control [36]. However, a meta-analysis of studies conducted in sub-Saharan Africa suggested that some couples may be cautious of testing together, preferring the option to test separately [37]. As such, offering home-based HIV testing may provide more acceptable testing circumstances for male partners, as discussed in the section below.

#### Conditional Economic Incentives

Conditional economic incentives can include monetary compensation and/or household goods in exchange for receipt of an HIV test. This approach has been used to successfully motivate HIV testing in many global settings. In Zimbabwe, groceries have successfully improved uptake of couples testing, from 10% in the non-incentivized group to 55% in the incentivized group [38]. Monetary incentives have also been successful among adolescents in Zimbabwe, more than doubling rates of facility-based HIV testing [39]. Similar findings were reported from a Kenyan study in which financial incentives increased the odds of adult PLWH bringing their children to facilities for HIV testing.

## Community-Based Strategies

Community-based HIV testing interventions aim to overcome well-described barriers to facility-based HIV testing such as anticipated stigma of seeking HIV testing at health facilities [40, 41], and structural barriers, such as poverty and poor health infrastructure that make accessing testing facilities difficult [42–44]. Community-based interventions have taken the form of home-based HIV testing, mobile HIV testing campaigns, hybrid approaches, partnerships with trusted community leaders, and social behavior change communication campaigns. Hybrid approaches are those that involve more than one modality of community-based testing and may incorporate both at-home self-testing and mobile HIV clinics.

### Home Based and Self Testing Interventions

Door-to-door home-based and self-testing interventions involve delivery of HIV testing to participants’ homes. Intervention strategies vary, with some having community health workers deliver or administer HIV tests, and others relying on family members or partners to deliver HIV self-tests. In a randomized controlled trial in Lesotho, intervention arm participants received HIV self-tests delivered to their homes if they were absent or refused door-to-door testing delivered by a health worker. This strategy improved HIV testing by 21%, with 81% of individual completing an HIV self-test compared with 60% in the referral group [45]. Data have suggested that home-based, self-testing methods may be particularly impactful among men [46, 47]. For example, in Zambia, a home-delivered HIV self-testing intervention resulted in higher testing among men, but not women [48••]. Similar successes were described in another intervention with HIV self-testing targeted to men and young adults in Malawi: 90% of eligible participants accepted HIV testing [49]. A study in rural South Africa found that home-based testing, coupled with financial incentives, improved uptake of HIV testing among men [50].

Other home-based strategies have sought to leverage the potential of women, who are already seeking care at facilities, through partner-delivered HIV self-testing kits. In Malawi, this approach improved uptake of HIV testing among male partners [51]. Similar results were found in randomized trials in Uganda and Kenya where women returning home from antenatal clinics delivered HIV self-testing kits to their male partners, increasing uptake of HIV testing three-fold compared with inviting men for clinic-based testing [52, 53]. Modeling strategies suggest that investing in scale up of home-based, self-testing strategies could expand coverage of HIV testing uptake to over 96% by 2030 in some countries [54].

### Mobile HIV Testing

Mobile HIV testing campaigns deliver HIV testing into communities via mobile clinics, reducing geographic barriers to accessing HIV testing services. Mobile campaigns have been shown to effectively reach priority populations, such as MSM and transgender women, in Thailand [55]. In South Africa, a targeted intervention for men coupled mobile testing with peer messaging about HIV treatment as a form of prevention. When compared to referral to mobile testing alone, peer messaging about treatment as prevention increased uptake of HIV testing from 13 to 22% [56•].

### Hybrid HIV testing

Hybrid approaches may vary in how they combine HIV testing modalities to maximize HIV testing uptake. Mobile hybrid strategies have been tremendously successful in low-income settings in East Africa, reaching up to 89% coverage of adults in some cases [57]. Hybrid approaches have also been effective at increasing HIV testing among adolescents [58]. One study found that a hybrid HIV testing intervention doubled the number of pediatric HIV infections identified in Kenya and Uganda [59]. Hybrid studies have also incorporated HIV testing as part of multi-disease screening campaigns. A multi-disease campaign in Uganda and Kenya improved HIV testing prevalence in communities from 57 to 90% in 1 year; after 3 years, HIV testing coverage was 98% in intervention communities compared with 96% in control communities [60••]. Another study in Uganda alone reported similar findings with 93% HIV testing uptake using a multi-disease campaign strategy [61].

### Partnerships with Trusted Community Leaders

In many parts of the world, traditional and faith healers are an integral part of local health systems. Eighty percent of the population in Africa and Asia rely on traditional medicine for their primary healthcare needs [62, 63], and 35% of Americans report using traditional medicines [64]. It is well-documented that PLWH engage regularly with traditional healers in sub-Saharan Africa [65–67], and many have suggested that traditional healers are a largely untapped resource to engage PLWH and improve HIV testing among rural populations [68–70]. In rural Mozambique, traditional healers effectively referred their clients to local clinics for HIV testing; however, only 5% of clients who presented to clinics actually received HIV testing [71]. In rural Uganda, a cluster randomized trial demonstrated that healer-facilitated HIV self-testing could quadruple the uptake of HIV testing, from 23% among those referred to facilities to 100% where self-testing was offered at the healer practice [72••]. These interventions are particularly noteworthy as they may be engaging rural populations that might otherwise not engage with biomedical care. In the USA, church-based HIV testing programs have been successful among Black and Latinx populations [73]. This may be a key avenue for improving HIV testing among underrepresented minority populations who bear a disproportionate burden of HIV [74].

### Social Behavior Change Communication

Cultural norms often serve as prominent barriers to HIV testing [75]. Consequently, some interventions have focused on social and behavioral change communication strategies as an avenue to improve uptake of HIV testing. In the Ivory Coast, an intervention targeting male gender norms—which included community-based HIV testing, guided group discussions, and male peer navigators—achieved 81% uptake of HIV testing among men [76]. Other programs have developed interventions focused on communication to reduce HIV-related stigma. For example, a stigma reduction intervention with African American and Latinx churches in the USA improved HIV testing [77].

## Strategies Targeting Priority Populations

Priority populations include MSM, transgender people, PWID, and people living in prisons/closed settings. Over 60% of new HIV transmissions occur among these priority populations and/or their partners and clients globally [9]. These populations require differentiated approaches for delivery of HIV testing due to stigmatization and/or criminalization [78]. Long distance truck drivers and adolescent girls and young women (AGYW) are also considered priority populations in sub-Saharan Africa. According to one systematic review, HIV self-testing interventions have improved uptake of HIV testing among priority populations by 1.45 times when compared to facility-based testing [79]. Interventions that target priority populations may leverage technological advances, such as mHealth, with delivery of HIV self-testing kits, to provide confidential and convenient HIV testing.

### Men Who Have Sex with Men and Transgender Populations

Strategies for improving HIV services uptake among MSM and transgender populations include outreach at venues that are frequented by these populations. In Sweden, rapid HIV tests offered at gay venues resulted in 96% testing uptake; many individuals reported they likely would not have received testing at a health facility [80]. In Thailand, HIV testing delivered at a gay sauna had 41% HIV testing uptake [81]. Peer-led support has also been effective in increasing testing among MSM. In a meta-analysis, interventions led by MSM peers have been shown to double the odds of receiving an HIV test [82]. Mobile health/mHealth interventions involve the use of mobile technologies and typically involve text messages, apps, or social media platforms to encourage HIV testing [83]. In China, mHealth Interventions have successfully linked MSM to HIV self-tests [84••, 85] and improved HIV testing frequency [86, 87]. Pilot studies have also begun to assess the potential for mHealth apps to improve uptake of HIV testing among adolescent MSM [88]. A randomized controlled trial among MSM in India found that 12 weeks of internet-based messaging on avoiding HIV acquisition resulted in higher uptake of HIV testing [89]. Finally, it is important to note that while HIV testing rates among MSM have improved in recent years, data from one meta-analysis among African MSM showed that countries with stricter anti-LGBTQ legislation had lower rates of HIV testing [90].

### People Who Inject Drugs

HIV testing rates are extremely low among PWID [91]. For example, despite free access to HIV testing at substance use disorder treatment programs, nearly one-third of PWID in the USA did not receive HIV testing [92]. In India, an integrated intervention among both MSM and PWID improved HIV testing uptake when compared to usual care, from 27 to 40% among MSM and from 25 to 34% among PWID. [93•]. Continued research is needed on novel interventions to improve uptake of HIV testing among PWID.

### Female Sex Workers

SMS text messages and peer educators delivering HIV self-testing have been shown to improve uptake of HIV testing among female sex workers. SMS text messages about HIV self-testing kits availability at a nearby wellness center nearly doubled odds of sex workers receiving HIV tests in Kenya [94]. Among Ugandan female sex workers, HIV self-testing coupled with peer-delivered education resulted in 23% more HIV testing where 95% of participants completed an HIV test compared with facility-based testing where 72% received an HIV test [95].

### Long Distance Truck Drivers

HIV testing interventions have had limited success among long distance truck drivers due to their mobility. In Kenya, one intervention used text messages to announce availability of HIV self-testing kits. This intervention doubled the uptake of HIV testing in the intervention group, but overall uptake of HIV testing was only 4% [96]. Another study conducted in Mozambique, South Africa, and Zimbabwe employed a similar technique and reported similar results among long distance truck drivers and female sex workers [97].

### Adolescent Girls and Young Women

An HIV testing campaign in Haiti that used community health workers to recruit AGYW demonstrated significant success, achieving 98% HIV testing uptake [98]. Delivering HIV testing via youth-friendly providers was also shown to increase the likelihood of receiving an HIV test among AGYW in Malawi [99].

## Ongoing Challenges

### Men Continue to be Missed by Current HIV Testing Programs

A 2019 study conducted in six sub-Saharan African countries found that approximately twice as many sexually active men had never been tested for HIV, compared with women. However, when offered an HIV test, the vast majority accepted testing for the first time [100]. This work underscores the fact that men in sub-Saharan Africa may be willing to undergo HIV testing if given the opportunity but are missed by existing programs. Future HIV testing interventions must consider factors that have been shown to shape HIV testing preferences—masculinity, economic priorities, and behaviors with risk of HIV acquisition—when developing interventions to effectively reach these populations.

### PWID

A recent meta-analysis suggested that PWID with access to needle exchange programs, or involvement in the criminal justice system, have increased likelihood of receiving an HIV test [101]. Outside of these institutional settings, PWID have extremely low uptake of HIV testing, despite very high acquisition risk. A study in the USA showed that PWID in low-income communities have lower HIV testing rates, compared with those living in higher income ones [102]. Targeted interventions are urgently needed to improve HIV testing among this priority population.

### PLWH Who Use Alternative Healing Systems

Many PLWH seek care from traditional healers or other informal healthcare providers, in place of or concurrently with biomedical care [103]. Additionally, some are dissuaded from seeking biomedical care due to perceived lapses in confidentiality, poor treatment, and difficulty accessing biomedical care [104–106]. Consequently, partnering with traditional health providers may be a key avenue for closing the gap in HIV testing among global populations, particularly among those reticent to engage with biomedical care more generally.

### Subsequent Steps of the HIV Continuum of Care

HIV testing is only the first step in the HIV continuum of care. Timely ART initiation is imperative for achieving viral suppression and preventing ongoing HIV transmissions. While community-based HIV testing programs have markedly increased uptake of HIV testing, entry into HIV care has been suboptimal following testing. In one home-based HIV testing strategy in Uganda and South Africa, only 50% of PLWH linked to care within 1 month [107]. In another South African study, large-scale HIV testing was not shown to reduce HIV incidence, likely due to poor linkage to care [108••]. It is therefore critical that future HIV testing interventions consider subsequent barriers to PLWH entering care once they are aware of their HIV status.

## Conclusions

Innovations in facility- and community-based HIV testing have brought us steps closer to achieving UNAIDS goal of epidemic control, starting with 95% of all PLWH being aware of their status. Despite this, current HIV testing programs are still missing hard-to-reach populations such as men in sub-Saharan Africa, people who inject drugs, and those who use alternative healing systems. In addition, interventions that have been shown to be effective in one setting may not be generalizable to a different cultural and social contexts; formative work is necessary to determine if interventions can be successfully adapted to other settings. Finally, it is critical that HIV testing programs consider effective approaches for linking those newly identified as living with HIV to care to prevent ongoing transmission.
